# GH peak response to GHRH-arginine: relationship to insulin resistance and other cardiovascular risk factors in a population of adults aged 50–90

**DOI:** 10.1111/j.1365-2265.2006.02569.x

**Published:** 2006-08

**Authors:** John D Carmichael, Ann Danoff, Daniela Milani, Ronenn Roubenoff, Martin L Lesser, Elayne Livote, Richard E Reitz, Steven Ferris, David L Kleinberg

**Affiliations:** * Neuroendocrine Unit, Division of Endocrinology, General Clinical Research Center, New York University School of Medicine and Department of Veterans Affairs Medical Center New York; † Nutrition, Exercise Physiology, and Sarcopenia Laboratory, Jean Mayer USDA Human Nutrition Research Center on Aging, Tufts University Boston, MA; ‡ Institute for Medical Research, North Shore LIJ Health System Manhasset, NY; § Quest Diagnostics Nichols Institute, San Juan Capistrano CA, USA; ¶ Alzheimer's Disease Center, New York University School of Medicine New York, NY, USA

## Abstract

**Objective:**

To assess the GH response to GHRH-arginine in apparently healthy adults in relation to cardiovascular risk factors.

**Design:**

Cross-sectional.

**Patients:**

Eighty-six male and female volunteers aged 50–90.

**Measurements:**

GH peak response to GHRH-arginine and cardiovascular risk factors, including obesity, insulin resistance, low levels of high density lipoprotein (HDL) cholesterol, elevated triglycerides, and hypertension. The primary outcome measurement was GH response to GHRH-arginine. The relationship between GH peak responses and cardiovascular risk factors was determined after data collection.

**Results:**

GH peaks were highly variable, ranging from 2·3 to 185 µg/l (14% with GH peaks < 9 µg/l). An increasing number of cardiovascular risk factors were associated with a lower mean GH peak (*P <* 0·0001). By univariate analysis, fasting glucose, insulin, body mass index (BMI), HDL cholesterol and triglycerides were significantly associated with GH peak (all *P* < 0·0001). Multiple regression analysis revealed that fasting glucose, fasting insulin, BMI, triglycerides and sex accounted for 54% of GH peak variability. The role of abdominal fat as it relates to GH peak was explored in a subset of 45 subjects. Trunk fat and abdominal subregion fat measured by dual energy X-ray absorptiometry (DXA) were inversely related to GH peak (*P* < 0·008 and 0·001, respectively). Analysis of this subgroup by multiple regression revealed that subregion abdominal fat became the significant obesity-related determinant of GH peak, but still lagged behind fasting insulin and glucose.

**Conclusions:**

GH response to secretagogues was highly variable in apparently healthy adults aged 50–90 years. Peak GH was significantly related to fasting glucose, insulin, BMI, HDL cholesterol, triglycerides, trunk fat and abdominal subregion fat, with fasting glucose ranking first by multiple regression analysis. There was a strong relationship between cardiovascular risk factors and low GH, with individual risk factors being additive. Although these data do not differentiate between low GH being a cause or an effect of these cardiovascular risk factors, they indicate that the relationship between low GH and increased cardiovascular risk may be physiologically important in the absence of pituitary disease.

## Introduction

Cardiovascular risk factors such as obesity, insulin resistance and dyslipidaemia cause major morbidity and mortality in the adult population.^1^,^2^ A similar association has been established in children and adults with GH deficiency (GHD) due to pituitary or hypothalamic disease, imparting increased morbidity and mortality largely due to cerebrovascular and cardiovascular disease.^3^–^7^ Treatment with GH has been demonstrated to improve cardiovascular risk profiles in subjects with GHD due to pituitary or hypothalamic disease.

Over the years, investigators have sought to establish the determinants of GH secretion in normal adults. Many relationships have been consistently demonstrated: lower 24 h GH secretion is associated with advancing age,^8^–^11^ male gender,^12^ obesity,^13^ total body fat^14^–^19^ and abdominal adiposity.^20^–^25^ Similarly, GH responses to a variety of pharmacological stimuli have shown an inverse relationship to body mass index (BMI)^13^,^25^–^32^ and abdominal adiposity.^33^,^34^ Some of these studies have also demonstrated relationships between GH secretory patterns and fasting insulin^24^ and serum lipids.^23^

While other studies have demonstrated relationships between GH dynamics and various cardiovascular risk factors in normal adults, GH response to secretagogues has not been assessed in a self-selected adult population of active, relatively healthy older individuals, nor has a relationship between GH peak and a clustering of cardiovascular risk factors been demonstrated. To determine normative data on GH response to GHRH-arginine, we studied a group of 86 apparently healthy adult male and female volunteers between the ages of 50 and 90. These responses were later analysed in relation to the presence or absence of cardiovascular risk factors.

## Methods

### Study subjects

To assess the variability of GH responses to GH secretagogues, we recruited a group of 86 normal volunteers between the ages of 50 and 90. This was initially done in collaboration with the Aging and Dementia Center at NYU and subsequently through town meetings, local advertisements and word of mouth. Volunteers were excluded from participating if they were nonambulatory, had a history of nonbasal cell malignancy, chronic renal or liver disease, cognitive or psychological dysfunction, were taking antipsychotic medications, or if they had been treated with GH during the 6 months prior to participation. None of the subjects, including those with BMI < 25, had undergone any recent significant weight changes. All subjects were screened for the presence of pituitary dysfunction by hormonal analysis. Subjects with pituitary abnormalities were excluded from the study.

### Study protocol

The NYU Institutional Board of Research Associates approved the protocol. After informed consent, each participant had a complete history and physical examination, electrocardiogram, fasting screening laboratory analysis, and endocrine function testing.

Two weeks after the initial visit, subjects underwent a repeat fasting measurement of IGF-I and GH stimulation testing with the combination of GHRH (1 µg/kg) and arginine (30 g in 300 ml over 30 min) infused intravenously. Samples were drawn through an indwelling catheter every 30 min from −30 min to 120 min, with subjects supine.

Cardiovascular risk factors were assessed in each individual and subjects were identified as having or not having abnormalities of five different categories of cardiovascular risk: increased BMI (≥ 27·5), low HDL [< 1·0 mmol/l (< 40 mg/dl) for males and < 1·3 mmol/l (< 50 mg/dl) for females], elevated triglycerides [≥ 1·7 mmol/l (≥ 150 mg/dl) or use of nonstatin lipid therapy], hypertension (history of hypertension or blood pressure ≥ 130/85 mmHg), and insulin resistance [history of type 2 diabetes mellitus or fasting glucose > 6·1 mmol/l (> 110 mg/dl)]. After identifying abnormalities of individual cardiovascular risk, the subjects were divided into two groups: those with two or fewer abnormal cardiovascular risk categories (group A) and those with three or more cardiovascular risk categories (group B).

All tests and measurements were carried out on the 86 volunteers except dual energy X-ray absorptiometry (DXA) scans, which were performed on a subset of 45 of the initial 86 volunteers. All subjects available and willing to undergo DXA scanning were included.

### Laboratory studies

Screening laboratory tests including complete blood count, hepatic panel, basic metabolic panel, serum cortisol, lipid profile and urinalysis were undertaken by standard techniques at the Bellevue Hospital Center Laboratory. Endocrine function testing, including TSH, free thyroxine, FSH, LH, oestradiol, PRL and total testosterone, was undertaken by Quest Diagnostics (Teterboro, NJ, USA) using standard techniques.

GH and IGF-I were measured by Quest Diagnostics Nichols Institute (San Juan Capistrano, CA, USA) using the Nichols Advantage® HGH and IGF-I assays. The GH assay is a two-site chemiluminescence immunoassay with a sensitivity of 0·1 µg/l. The intra-assay coefficients of variation (CVs) were 4·2%, 4·8% and 8% for detected GH levels at 0·8 µg/l, 2·42 µg/l and 23·6 µg/l, respectively. The interassay CVs were 4·1%, 5·8% and 12·1% with corresponding GH levels of 0·92 µg/l, 2·40 µg/l and 23·7 µg/l. Methods for the chemiluminescent assay for IGF-I have been reported previously.^35^ Total serum insulin was measured by radioimmunoassay (RIA) after acid treatment and polyethylene glycol (PEG) precipitation (Quest Diagnostics Nichols Institute).

The homeostatic model assessment of insulin resistance (HOMA-IR) was calculated using fasting insulin levels (mU/l) and simultaneous fasting serum glucose (mmol/l) using the following equation: HOMA-IR = (fasting insulin × fasting glucose)/22·5.^36^

### Body composition measurement

Forty-five subjects completed DXA scanning. Whole-body DXA measurements were obtained using the Hologic QDR-1000/W (Waltham, MA, USA) with standard protocol. The same experienced technician performed all of the scans. Although scans were obtained 2 years after the initial workup, the weights obtained at the time of DXA scanning were highly correlated with those obtained at baseline (*r* = 0·973, *P* < 0·0001). We analysed the QDR-1000/W results for the whole body as well as for regions of interest, including the trunk, arms and legs. During a separate analysis, a manually defined abdominal subregion was examined by DXA.^37^ Measurements of total body fat, trunk fat and abdominal subregion fat were used for this analysis. Total body DXA has a CV of 2–3% for total body fat mass and 1–2% for fat free mass.^38^,^39^ Measures of trunk fat and abdominal fat by DXA have been found to correlate significantly with measures of total abdominal fat and abdominal visceral fat by computed tomography (CT).^37^,^40^ Trunk fat and abdominal subregion fat were corrected for total body fat and expressed as the ratio of trunk or subregion to total body fat.^41^

### Statistics

The first objective of the statistical analysis was to determine which variables, alone or in combination, were associated with GH peak. Prior to performing a multiple regression analysis, candidate predictor variables were identified by using simple correlation analysis for each predictor variable with the log GH peak. Those variables with correlations significant at *P* < 0·05 (BMI, HDL cholesterol, triglycerides, total/HDL cholesterol ratio, fasting glucose, fasting insulin and sex) were entered into the regression model. Multiple regression analysis using a backward elimination method was applied to the data with *P* < 0·10 being the criterion for a variable to remain in the model. BMI was used as the preferred method over weight as a measure of obesity in the regression model. A second multiple regression analysis was performed on the subset of 45 subjects who returned for whole-body DXA scan. Methods for this multiple regression model were identical to those described above. Those variables with correlations significant at *P* < 0·05 (BMI, triglycerides, HDL cholesterol, total/HDL cholesterol ratio, fasting glucose, fasting insulin, and corrected trunk fat and corrected abdominal subregion fat) were entered into this regression model. The percentage of contribution to the variance in GH peak for each individual parameter was determined by using the square of the Pearson correlation coefficient (*R*^2^). Standardized regression coefficients were used to rank the variables that remained in the final regression models. Comparison of means was performed using Student's *t*-test, unless noted otherwise. One-way analysis of variance (anova) was used to compare mean log GH peak values according to the number of cardiovascular risk parameters within subjects. Analyses were performed using SAS version 9·1 (SAS Institute, Cary, NC, USA). All analyses involving GH peak were performed on base 10 log transformed GH peak responses to GHRH-arginine secretagogue testing because the log transform yielded nearly gaussian residuals in the regression model. However, for simplicity of presentation, descriptive statistics regarding GH peak are presented in original untransformed units, and have been plotted using a logarithmic scale.

## Results

### Demographics

Demographic data are presented in Table 1. There were 57 women and 29 men: 82·5% of the subjects were non-Hispanic Caucasian, 12·8% Hispanic Caucasian, 3·5% Asian, and 1·2% Black. Mean age was 65·4 ± 9·1 years [all data are presented as mean ± standard deviation (SD) unless noted otherwise]. BMI ranged from 19·3 to 48·5. Thirty-five per cent of subjects had BMI < 25, 41% ≥ 25 and < 30, 21% ≥ 30 and < 40, and 4% ≥ 40. Mean IGF-I for the sample was 122·8 ± 52·2 µg/l at visit 1 and 119·4 ± 52·6 µg/l at visit 2 (1 µg/l = 1 ng/ml). The variability and reliability of a single IGF-I measurement in these volunteers has been reported previously.^35^

**Table 1:** Demographics for the whole group and the subset that had subsequent DXA scans

	Whole group (*n* = 86)	DXA subset (*n* = 45)	Significance
Gender	57F/29M	32F/13M	ns*
Age (years)	65·4 ± 9·2	64·0 ± 10·1	ns*
BMI (kg/m^2^)	27·6 ± 5·6	27·0 ± 5·4	ns*
Height (cm)	163·9 ± 9·8	164·3 ± 9·3	ns*
Weight (kg)	74·5 ± 17·7	73·2 ± 17·7	ns*
Fasting glucose (mmol/l)	5·4 ± 1·2	5·2 ± 0·6	ns*
Systolic BP (mmHg)	129·3 ± 17·8	128·0 ± 17·8	ns*
Diastolic BP (mmHg)	73·6 ± 10·6	73·1 ± 10·1	ns*
Total cholesterol (mmol/l)	5·3 ± 1·2	5·6 ± 1·1	ns*
HDL cholesterol (mmol/l)	1·6 ± 0·5	1·6 ± 0·5	ns*
LDL cholesterol (mmol/l)	3·2 ± 1·0	3·4 ± 0·8	ns*
Triglycerides (mmol/l)	1·5 ± 0·8	1·5 ± 1·0	ns*
HOMA-IR	3·6 ± 2·6	3·0 ± 1·5	ns*
GH peak (µg/l)			
Arithmetic mean (± SD)	34·8 ± 35·9	39·7 ± 41·5	ns†
Median	23·2	31·3	
IGF-I (µg/l)			
Visit 1	122·8 ± 52·2	129·1 ± 59·5	ns*
Visit 2	119·4 ± 52·6	126·9 ± 62·1	ns*
Coronary artery disease (*n*)	4	3	ns‡
Diabetes mellitus type 2 (*n*)	7	2	ns‡
Hyperlipidaemia (*n*)	29	15	ns‡
Hypertension (*n*)	24	13	ns‡
Lipid therapy (*n*)	17	8	ns‡
Oral diabetes therapy (*n*)	5	1	ns‡
Hypertension therapy (*n*)	22	11	ns‡
BMI ≥ 27·5 (*n*, %)	42 (49)	19 (42)	ns§

DXA, dual energy X-ray absorptiometry; BP, blood pressure; HDL, high density lipoprotein; LDL, low density lipoprotein; BMI, body mass index.All data presented as mean ± standard deviation unless noted otherwise.ns = P > 0·05 by *Student's t-test, †Student's t-test using mean log GH, ‡Fisher's exact test, and §χ^2^.

18 mg/dl glucose = 1 mmol/l; 38·67 mg/dl cholesterol = 1 mmol/l; 88·57 mg/dl triglycerides = 1 mmol/l.

### Response of GH to GHRH-arginine stimulation

**Fig. 1:**
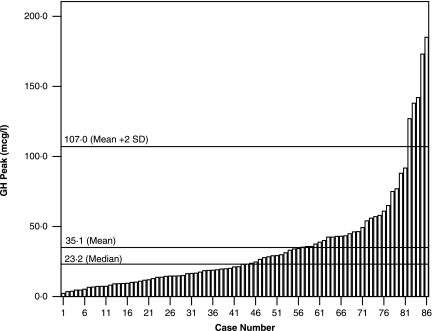
Variability of peak GH responses to GHRH-arginine. Individual peak GH responses in 86 volunteers between 50 and 90 years of age.

GH peak varied from 2·3 to 185 µg/l (mean 34·8 ± 35·9 µg/l; median 23·2 µg/l; Fig. 1). Five volunteers had values consistent with a rigorous definition of GHD (peak GH < 5·0 µg/l).^42^ An additional seven individuals had GH peaks between 5·0 and 8·2 µg/l, values consistent with GHD by other criteria (GH ≤ 9·0 µg/l).^43^,^44^ Twenty-nine subjects had GH peak responses less than 16 µg/l, the cutoff point suggested by Ghigo *et al.* for normal subjects.^45^ Thus, while not diagnostic of GHD, 12 subjects (14%) of our sample population had GH peaks that would be consistent with GHD (< 9 µg/l).^43^

GH peaks were above 107·0 µg/l (> 2 SD above mean peak) in five volunteers. All were female and none had elevated IGF-I levels or clinical features suggestive of acromegaly. All were postmenopausal and four of the five were taking hormone replacement therapy. One subject was taking a daily oral conjugated oestrogen–progesterone combination tablet, one a weekly oestradiol patch, one a biweekly combination oestradiol–norethindrone acetate patch, and one was taking oral medroxyprogesterone and using topical oestrogen cream. Mean BMI was 21·2 ± 1·8 (all < 25) and mean height was 155 ± 6·3 cm (range 148–160 cm). We did not consider them GH resistant because nonstimulated GH levels were not elevated (GH t-30 and GH t0 means were 2·76 ± 2·8 and 0·98 ± 0·932 µg/l, respectively).

One female subject had a GH peak of 3·5 µg/l and a confirmed, elevated IGF-I of 351 µg/l. She was fully evaluated and found to have a normal pituitary magnetic resonance imaging (MRI), normal GH suppression in response to oral glucose and no physical signs or symptoms consistent with acromegaly. No explanation for this disparity between GH results and IGF-I was found. Despite her abnormal results, she met inclusion and exclusion criteria and was therefore included in the analyses.

### Relationship of GH peak to age and gender

The relationship between GH peak responses to GHRH-arginine and age was not significant. As reported previously,^12^ the mean GH peak was higher in women than in men (41·7 ± 41·2 *vs.* 21·2 ± 14·8 µg/l, respectively; *P* < 0·01). This difference was not attributable to BMI, which was not significantly different between men and women. Three female subjects were not postmenopausal and 13 postmenopausal women were taking exogenous oestrogen. When the mean of log GH peak of postmenopausal women not on oestrogen (*n* = 41) was compared to that of men (*n* = 29), the difference was still significant (*P <* 0·002). For male subjects, there was a significant direct relationship between log GH peak and testosterone levels (*r* = 0·562, *P* < 0·002). For male or female subjects, the correlation between log GH peak and oestrogen levels was not significant.

### Relationship of individual cardiovascular risk factors to GH peak

There were significant correlations between log transformed GH peak and BMI, HDL, triglycerides, cholesterol/HDL ratio, fasting glucose, fasting insulin, and HOMA-IR (Table 2).

**Table 2:** Correlation between log GH peak and parameters of cardiovascular risk

	Whole group (*n* = 86)		BMI ≥ 27·5 (*n* = 42)		DXA subset (*n* = 45)	
			
Parameter	*r*	*P*	*r*	*P*	*r*	*P*
BMI	–0·51	< 0·0001	–0·18	< 0·253	–0·57	< 0·0001
HDL cholesterol	0·43	< 0·0001	0·22	< 0·168	0·35	< 0·02
Triglycerides	–0·41	< 0·0001	–0·21	< 0·192	–0·39	< 0·008
Fasting glucose	–0·55	< 0·0001	–0·50	< 0·001	–0·61	< 0·0001
Systolic BP	–0·16	< 0·152	0·12	< 0·433	–0·07	< 0·649
Diastolic BP	–0·14	< 0·20	–0·08	< 0·638	–0·05	< 0·736
Insulin	–0·52	< 0·0001	–0·41	< 0·007	–0·65	< 0·0001
HOMA-IR	–0·52	< 0·0001	–0·43	< 0·005	–0·69	< 0·0001
Chol/HDL	–0·44	< 0·0001	–0·18	< 0·268	–0·45	< 0·002
Total cholesterol	0·06	< 0·58	0·15	< 0·329	0·01	< 0·976
LDL cholesterol	–0·01	< 0·96	0·16	< 0·317	–0·03	< 0·847

BMI, body mass index; DXA, dual energy X-ray absorptiometry; HDL, high density lipoprotein; BP, blood pressure; HOMA-IR, homeostasis model assessment – insulin resistance; Chol/HDL, total cholesterol/high density lipoprotein cholesterol; LDL, low density lipoprotein. Pearson correlation coefficients between parameters and log GH peak for the whole group (n = 86), a subgroup of 42 subjects whose BMI was ≥ 27·5, and a subgroup of 45 subjects having had body composition measurements by DXA. Significant relationships are shown in bold.

### Studies relating to clustering of cardiovascular risk factors

**Fig. 2:**
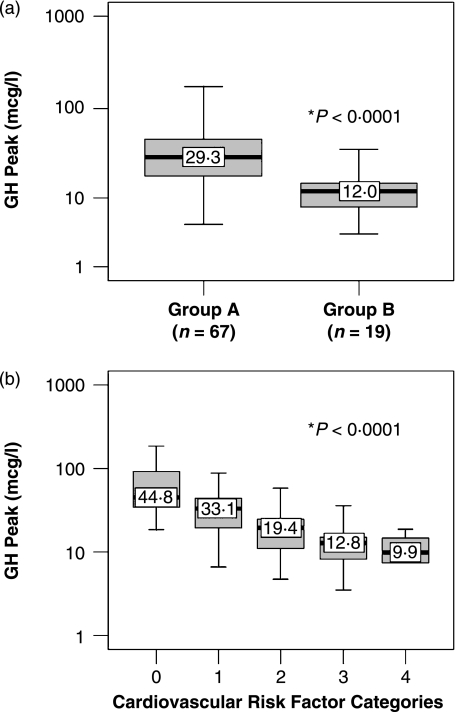
Relationship of GH peak to cardiovascular risk factors. (a) Mean log GH peak was significantly lower in 19 volunteers with three or more cardiovascular risk factors compared to 67 with two or fewer cardiovascular risk factors. (b) GH peak in 86 subjects divided according to number of cardiovascular risk factors [0 (*n* = 22), 1 (*n* = 23), 2 (*n* = 22), 3 (*n* = 13), and 4 (*n* = 6)]. Median arithmetic values for GH peak are displayed. Statistics performed by Student's *t*-test (a) and one-way analysis of variance (anova) (b). (1 ng/ml GH = 1 µg/l.)

Subjects were identified as having or not having an abnormality of the five following cardiovascular risk factors: obesity, hypertension, low HDL, high triglycerides, and insulin resistance. Twenty-two subjects had no abnormal risk factors, 23 had one of five categories, 22 had two of five categories, 13 had three of five, and six had four of five categories. None had all five categories. Subjects were then grouped according to the number of abnormal categories: group A (two or fewer categories) and group B (three or more abnormal cardiovascular risk factors). Nineteen of the 86 subjects (22%) were in group B and 67 of 86 (78%) were in group A. Mean log GH peak was significantly lower in subjects in group B than in those in group A (*P <* 0·0001; Fig. 2a). When subjects were assessed according to the number of cardiovascular risk factors, one-way anova of mean log GH peaks according to the number of cardiovascular risk categories (0, 1, 2, 3 and 4 risk factors) revealed a significant difference between groups. An increasing number of risk factor categories was associated with lower GH peaks (*P <* 0·0001; Fig. 2b).

### Multiple regression analysis

Candidate variables for multiple regression analysis were determined based on significance of factors related to GH peak determined by univariate analysis as shown in Table 2. Fasting glucose, fasting insulin, BMI, triglycerides and sex explained 54% (*R*^2^ = 0·5379) of the variation in GH peak (Table 3). Based on the standardized regression coefficients, the relative importance of the five predictor variables from highest to lowest was: fasting glucose, BMI, triglycerides, sex and fasting insulin. Low GH peak was associated with high fasting glucose levels, high BMI, high triglycerides, male gender and high fasting insulin levels. Although HOMA-IR was highly correlated with GH peak, due to multicollinearity among HOMA-IR, insulin and glucose (because of the dependence of HOMA-IR on the other two variables), HOMA-IR was not used in the multiple regression analysis.

**Table 3:** Results of multiple regression analysis

Variable	Coefficient	Standardized coefficient	Significance
Intercept	2·6129	0	< 0·0001
Fasting glucose	−0·0053	−0·2812	0·0030
BMI	−0·0190	−0·2782	0·0032
Triglycerides	−0·0019	−0·2654	0·0013
Sex	0·1770	0·2112	0·0134
Fasting insulin	−0·0096	−0·1636	0·0929

Analysis of the whole group (n = 86) showed that fasting glucose, body mass index (BMI), triglycerides, sex and fasting insulin accounted for 54% of GH variance.

### Relationship of BMI to GH peak

**Fig. 3:**
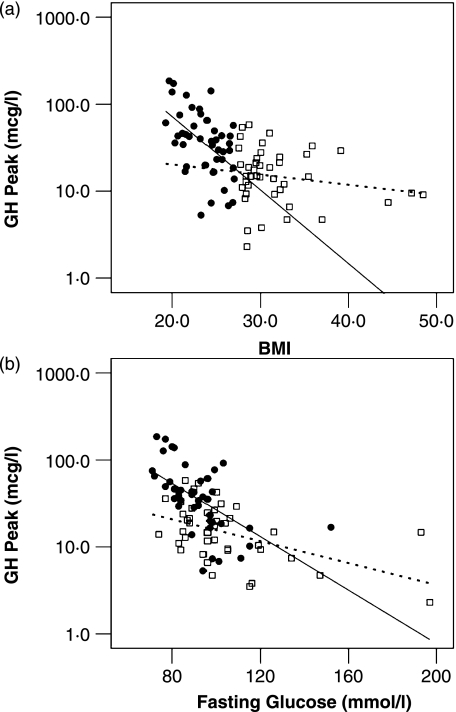
(a) Relationship of body mass index (BMI in kg/m^2^) to GH peak. Subjects with BMI < 27·5 are represented by filled circles (•) and those with BMI ≥ 27·5 by open squares (□). Separate regression lines were drawn for subjects with BMI < 27·5 (solid line) and those with BMI ≥ 27·5 (dashed line). Relationship between GH and BMI was significant when BMI was < 27·5 (*r* = –0·516, *P* < 0·0001) but was lost when only those with BMI ≥ 27·5 were considered (*r* = –0·181, *P* < 0·253). (b) Relationship of fasting glucose to GH peak according to BMI. For BMI < 27·5 the relationship between GH and fasting glucose was significant (*r* = –0·580, *P* < 0·0001), as was the relationship between GH and glucose in BMI ≥ 27·5 (*r* = –0·495, *P* < 0·001). (1 ng/ml GH = 1 µg/l.)

When the group as a whole was divided into subgroups according to BMI (≥ 27·5 and < 27·5), the relationship between BMI and GH peak was maintained in the lower BMI group but not in subjects with BMI ≥ 27·5 (Fig. 3a). By contrast, the relationships between GH peak and fasting glucose, fasting insulin and HOMA were maintained in both groups (Table 2, Fig. 3b, data not shown for fasting insulin and HOMA).

In the subgroup of subjects with BMI ≥ 27·5, variability in GH peak was still present. Mean log GH peak for subjects in group B (mean 12·7 ± 8·9 µg/l, median 11·2 µg/l, *n* = 18) was lower than for those in group A (mean 23·6 ± 14·4 µg/l, median 20·7 µg/l, *n* = 24) (*P <* 0·004). There was no statistically significant difference in BMI between subjects in group B *vs.* group A (30·9 ± 4·0 *vs.* 32·4 ± 5·7 kg/m^2^, respectively; *P* < 0·33).

### Assessment of abdominal adiposity

We measured total, trunk and abdominal subregion fat using whole-body DXA in a subset of 45 of the 86 original subjects. Measurements of abdominal subregion fat are highly correlated with measures of visceral adiposity by CT scan.^37^,^40^ There were no significant demographic differences between the whole group and the DXA participant group (Table 1), nor did they differ regarding their relationships between GH peak and cardiovascular risk factors (Table 2). In fact, weights obtained at the time of DXA scanning were highly correlated with those obtained at baseline (*r* = 0·973, *P* < 0·0001). Total body fat was significantly correlated with both trunk fat (*r* = 0·887, *P* < 0·0001) and abdominal subregion fat (*r* = 0·837, *P* < 0·0001). Total body fat was not significantly related to GH peak. However, corrected trunk fat (*r* = *–*0·606, *P* < 0·0001) and corrected abdominal subregion fat (*r* = *–*0·612, *P* < 0·0001) were associated with the log of GH peak. In each case, total body fat, trunk and abdominal subregion fat correlated with BMI (*P* < 0·0001).

### Subgroup multiple regression analysis

Multiple regression analysis was performed on the subgroup of 45 subjects who had body composition analysis by DXA. Candidate predictor variables were again determined based on the significance of factors related to the GH peak determined by univariate analysis as shown in Table 2. Total fasting insulin, fasting glucose, HDL and the corrected subregion fat explained 69% (*R*^2^ = 0·69) of the variation in GH peak. Based on the standardized regression coefficients, the relative importance of the predictor variables from highest to lowest was: fasting insulin, fasting glucose, HDL cholesterol and corrected subregion fat. Low GH peak was associated with high fasting insulin levels, high fasting glucose, low HDL and a high ratio of abdominal subregion fat to total body fat.

## Discussion

This paper presents the results of GH responses to GHRH-arginine in a group of 86 normal volunteers between the ages of 50 and 90. The study subjects were self-selected and were included on the basis of age, apparent health, and absence of pituitary disease. Their demographic profile with regard to obesity^46^ is comparable to that of the US population, suggesting that these results may be generalizable.

We noted a wide variation in the peak GH responses, which ranged from 2·3 to 185 µg/l. We found that 14% of the sample had GH peaks of 8·2 µg/l or below and 6% had values that were greater than 2 SD above the mean.

Our approach differs from previous studies assessing GH response to GHRH-arginine in a number of ways. Other studies selected their subjects based on the presence or absence of obesity,26–28,45 focused on younger age^47^ or as matched controls for the subjects with pituitary disease.^26^,^42^ It was concluded from these studies of selected subjects, as well as others measuring 24-h integrated GH concentration,^14^–^24^,^48^–^50^ that GH was low in obese subjects. Many studies demonstrated low GH concentrations or peaks in obese as compared to nonobese subjects.^14^,^25^,^32^,^48^ The observation that weight loss was associated with an increase in GH secretion adds support to the idea that obesity suppresses GH secretion.^15^,^32^,^51^ Although we also noted a significant correlation between BMI and log GH peak in the sample as a whole, we were surprised to find that the relationship between the two lost significance when subjects whose BMI was ≥ 27·5 were considered separately (*r* = *–*0·181, *P* < 0·253). The relationship between GH peak and BMI was maintained and strongly correlated in subjects with BMI below 27·5 (*P <* 0·0001; see Fig. 3a). Thus, we could conclude that leanness is a more important determinant of GH responsiveness than obesity is to impaired GH response. While other studies have not reported similar findings, Bonert *et al*. and Corneli *et al*. presented graphs on GH peak according to BMI that look very similar to ours.^26^,^28^ It is possible that GH peak does not go down further once a threshold for obesity is reached.

More recent studies comparing abdominal fat to GH dynamics by DXA,^21^ including the present study, or abdominal visceral fat by CT or MRI^22^–^24^,^33^,^34^ show a high degree of correlation between lower GH and higher abdominal fat. In this study, when we looked at abdominal fat in a subset of the large sample, abdominal subregion fat was a more significant predictor of low peak GH than high BMI.

Although the GHRH-arginine stimulation test is a valid measure of GH secretion in patients with pituitary disease, and does discriminate between GH-deficient and GH-sufficient individuals, the test does not necessarily reflect physiological GH secretion in subjects without pituitary disease. We observed no relationship between age and GH peak in response to GHRH-arginine stimulation. Other investigators have also noted the lack of relationship between age and GH peak with the use of this secretagogue test.^45^,^52^,^53^ The decline in GH secretion has been shown primarily with 24-h assessments of GH secretion, which may more accurately reflect physiological secretion. It is also important to note that the decline in GH secretion is not linear, with more sizeable reduction in secretion noted in the fourth and fifth decades of life.^50^ We observed an inverse relationship between GH peak and BMI, trunk fat and abdominal fat, independent of age in subjects between the ages of 50 and 90. While the GHRH-arginine test may demonstrate a preservation of GH secretory potential with age, it is still subject to the influences of BMI.

The inverse relationship between measures of insulin resistance and GH response was highly significant in univariate analyses and also ranked highest among significant contributors in the multiple regression analyses. In fact, when assessed according to BMI subgroup, the relationship was significant in subjects with both low and high BMIs. As far as we know, only in the study by Clasey *et al.* did fasting insulin play so important a role in GH secretory dynamics.^24^ Some have suggested a direct inhibitory effect of insulin on GH secretion.^54^,^55^

We also found a direct correlation between low GH peak and HDL cholesterol and an inverse relationship with triglycerides and the cholesterol/HDL ratio. This confirms the observation by O’Connor *et al*. who studied 24-h GH secretory patterns as they relate to lipids and abdominal fat in a similar group of subjects.^23^ They did not, however, assess measures of insulin resistance.

We found no correlation between the log of peak GH and low density lipoprotein (LDL) or total cholesterol, as well as no correlation between GH and measures of blood pressure. It should be noted that these parameters were evaluated in the presence of LDL targeted lipid therapy and hypertension therapy in many subjects, making the interpretation of direct correlations between GH and blood pressure, LDL or total cholesterol less reliable.

Although there are many common features between subjects with GHD due to pituitary disease and subjects with a clustering of cardiovascular risk factors in the absence of pituitary disease, it is not clear whether the low GH associated with these cardiovascular risk factors is cause or effect. Low GH in the setting of obesity has been shown to be reversed with weight reduction, implying that it is obesity that causes the low GH.^15^,^32^,^51^ By contrast, the observation that subjects with GHD due to pituitary or hypothalamic disease develop obesity and its metabolic consequences suggests that GH plays a physiological role in the development of obesity and its cardiovascular complications.^3^,^4^ This distinction is important in our understanding of the role of GH in the development and maintenance of cardiovascular risk factors, and may have therapeutic implications. A future study establishing the prevalence of low GH, determining the relationship of low GH to the various cardiovascular risk factors, and examining the efficacy of low-dose treatment with GH on improving cardiovascular risk factors may show the efficacy of GH treatment on improving these risk factors, but would still not prove cause or effect. One possible way of determining this distinction is by assessing subjects’ baseline GH status and following these subjects in a longitudinal trial, observing cardiovascular outcome over many years.

While previous studies have demonstrated a relationship between GH and individual cardiovascular risk factors in otherwise normal adults, our study is the first to show that peak GH is inversely proportional to the number of major cardiovascular risk categories. By studying a cohort of individuals largely representative of middle-class Caucasian New Yorkers, and assessing cardiovascular risk factors as part of the study rather than as inclusion criteria, we were able to examine differences between GH dynamics in those with cardiovascular risk factors compared to those without. The striking correlation of low GH to individual cardiovascular risk factors suggests an important role for GH in the clustering of these risk factors, whether or not the relationship is a causative one. Analogous to the gradual acceptance of a wide variety of what are now considered traditional cardiovascular risk factors, perhaps it is time to consider low GH as an independent marker for cardiovascular disease.

## References

[1] Reaven GM (2003). Insulin resistance/compensatory hyperinsulinemia, essential hypertension, and cardiovascular disease. Journal of Clinical Endocrinology and Metabolism.

[2] Isomaa B, Almgren P, Tuomi T, Forsen B, Lahti K, Nissen M, Taskinen MR, Groop L (2001). Cardiovascular morbidity and mortality associated with the metabolic syndrome. Diabetes Care.

[3] Newman CB, Kleinberg DL (1998). Adult growth hormone deficiency. Endocrinologist.

[4] de Boer H, Blok GJ, van der Veen EA (1995). Clinical aspects of growth hormone deficiency in adults. ENDO Review.

[5] Markussis V, Beshyah SA, Fisher C, Sharp P, Nicolaides AN, Johnston DG (1992). Detection of premature atherosclerosis by high-resolution ultrasonography in symptom-free hypopituitary adults. Lancet.

[6] Rosen T, Bengtsson BA (1990). Premature mortality due to cardiovascular disease in hypopituitarism. Lancet.

[7] Bengtsson B, Eden S, Lonn L, Kvist H, Stokland A, Lindstedt G, Bosaeus I, Tolli J, Sjostrom L, Isaksson OG.P (1993). Treatment of adults with growth hormone (GH) deficiency with recombinant human GH. Journal of Clinical Endocrinology and Metabolism.

[8] Rudman D, Kutner MH, Rogers CM, Lubin MF, Fleming GA, Bain RP (1981). Impaired growth hormone secretion in the adult population. Relation to age and adiposity. Journal of Clinical Investigation.

[9] Zadik Z, Chalew SA, McCarter RJ, Meistas M, Kowarski AA (1985). The influence of age on the 24-hour integrated concentration of growth hormone in normal individuals. Journal of Clinical Endocrinology and Metabolism.

[10] Finkelstein JW, Roffwarg HP, Boyar RM, Kream J, Hellman L (1972). Age-related change in the twenty-four-hour spontaneous secretion of growth hormone. Journal of Clinical Endocrinology and Metabolism.

[11] Iranmanesh A, Lizarralde G, Veldhuis JD (1991). Age and relative adiposity are specific negative determinants of the frequency and amplitude of growth hormone (GH) secretory bursts and the half-life of endogenous GH in healthy men. Journal of Clinical Endocrinology and Metabolism.

[12] Frantz AG, Rabkin MT (1965). Effects of estrogen and sex difference on secretion of human growth hormone. Journal of Clinical Endocrinology and Metabolism.

[13] De Marinis L, Folli G, D’Amico C, Mancini A, Sambo P, Tofani A, Oradei A, Barbarino A (1988). Differential effects of feeding on the ultradian variation of the growth hormone (GH) response to GH-releasing hormone in normal subjects and patients with obesity and anorexia nervosa. Journal of Clinical Endocrinology and Metabolism.

[14] Meistas MT, Foster GV, Margolis S, Kowarski AA (1982). Integrated concentrations of growth hormone, insulin, C-peptide and prolactin in human obesity. Metabolism.

[15] Rasmussen MH, Hvidberg A, Juul A, Main KM, Gotfredsen A, Skakkebaek NE, Hilsted J, Skakkebae NE (1995). Massive weight loss restores 24-hour growth hormone release profiles and serum insulin-like growth factor-I levels in obese subjects. Journal of Clinical Endocrinology and Metabolism.

[16] Veldhuis JD, Liem AY, South S, Weltman A, Weltman J, Clemmons DA, Abbott R, Mulligan T, Johnson ML, Pincus S (1995). Differential impact of age, sex steroid hormones, and obesity on basal versus pulsatile growth hormone secretion in men as assessed in an ultrasensitive chemiluminescence assay. Journal of Clinical Endocrinology and Metabolism.

[17] Iranmanesh A, Lizarralde G, Veldhuis JD (1991). Age and relative adiposity are specific negative determinants of the frequency and amplitude of growth hormone (GH) secretory bursts and the half-life of endogenous GH in healthy men. Journal of Clinical Endocrinology and Metabolism.

[18] Roubenoff R, Rall LC, Veldhuis JD, Kehayias JJ, Rosen C, Nicolson M, Lundgren N, Reichlin S (1998). The relationship between growth hormone kinetics and sarcopenia in postmenopausal women: the role of fat mass and leptin. Journal of Clinical Endocrinology and Metabolism.

[19] Weltman A, Weltman JY, Hartman ML, Abbott RD, Rogol AD, Evans WS, Veldhuis JD (1994). Relationship between age, percentage body fat, fitness, and 24-hour growth hormone release in healthy young adults: effects of gender. Journal of Clinical Endocrinology and Metabolism.

[20] Vahl N, Jorgensen JO, Skjaerbaek C, Veldhuis JD, Orskov H, Christiansen JS (1997). Abdominal adiposity rather than age and sex predicts mass and regularity of GH secretion in healthy adults. American Journal of Physiology.

[21] Miller KK, Biller BM, Lipman JG, Bradwin G, Rifai N, Klibanski A (2005). Truncal adiposity, relative growth hormone deficiency, and cardiovascular risk. Journal of Clinical Endocrinology and Metabolism.

[22] Pijl H, Langendonk JG, Burggraaf J, Frolich M, Cohen AF, Veldhuis JD, Meinders AE (2001). Altered neuroregulation of GH secretion in viscerally obese premenopausal women. Journal of Clinical Endocrinology and Metabolism.

[23] O’Connor KG, Harman SM, Stevens TE, Jayme JJ, Bellantoni MF, Busby-Whitehead MJ, Christmas C, Munzer T, Tobin JD, Roy TA, Cottrell E, St Clair C, Pabst KM, Blackman MR (1999). Interrelationships of spontaneous growth hormone axis activity, body fat, and serum lipids in healthy elderly women and men. Metabolism.

[24] Clasey JL, Weltman A, Patrie J, Weltman JY, Pezzoli S, Bouchard C, Thorner MO, Hartman ML (2001). Abdominal visceral fat and fasting insulin are important predictors of 24-hour GH release independent of age, gender, and other physiological factors. Journal of Clinical Endocrinology and Metabolism.

[25] Buijs MM, Burggraaf J, Wijbrandts C, de Kam ML, Frolich M, Cohen AF, Romijn JA, Sauerwein HP, Meinders AE, Pijl H (2003). Blunted lipolytic response to fasting in abdominally obese women: evidence for involvement of hyposomatotropism. American Journal of Clinical Nutrition.

[26] Corneli G, Di Somma C, Baldelli R, Rovere S, Gasco V, Croce CG, Grottoli S, Maccario M, Colao A, Lombardi G, Ghigo E, Camanni F, Aimaretti G (2005). The cut-off limits of the GH response to GH-releasing hormone-arginine test related to body mass index. European Journal of Endocrinology.

[27] Maccario M, Valetto MR, Savio P, Aimaretti G, Baffoni C, Procopio M, Grottoli S, Oleandri SE, Arvat E, Ghigo E (1997). Maximal secretory capacity of somatotrope cells in obesity: comparison with GH deficiency. International Journal of Obesity and Related Metabolic Disorders.

[28] Bonert VS, Elashoff JD, Barnett P, Melmed S (2004). Body mass index determines evoked growth hormone (GH) responsiveness in normal healthy male subjects: diagnostic caveat for adult GH deficiency. Journal of Clinical Endocrinology and Metabolism.

[29] Maccario M, Gauna C, Procopio M, Di Vito L, Rossetto R, Oleandri SE, Grottoli S, Ganzaroli C, Aimaretti G, Ghigo E (1999). Assessment of GH/IGF-I axis in obesity by evaluation of IGF-I levels and the GH response to GHRH+arginine test. Journal of Endocrinological Investigation.

[30] Kirk SE, Gertz BJ, Schneider SH, Hartman ML, Pezzoli SS, Wittreich JM, Krupa DA, Seibold JR, Thorner MO (1997). Effect of obesity and feeding on the growth hormone (GH) response to the GH secretagogue L-692,429 in young men. Journal of Clinical Endocrinology and Metabolism.

[31] Copinschi G, Wegienka LC, Hane S, Forsham PH (1967). Effect of arginine on serum levels of insulin and growth hormone in obese subjects. Metabolism.

[32] Williams T, Berelowitz M, Joffe SN, Thorner MO, Rivier J, Vale W, Frohman LA (1984). Impaired growth hormone responses to growth hormone-releasing factor in obesity. New England Journal of Medicine.

[33] Vahl N, Jorgensen JO.L, Jurik AG, Christiansen J (1996). Abdominal adiposity and physical fitness are major determinants of the age associated decline in stimulated GH secretion in healthy adults. Journal of Clinical Endocrinology and Metabolism.

[34] Erickson D, Keenan DM, Farhy L, Mielke K, Bowers CY, Veldhuis JD (2005). Determinants of dual secretagogue drive of burst-like growth hormone secretion in premenopausal women studied under a selective estradiol clamp. Journal of Clinical Endocrinology and Metabolism.

[35] Milani D, Carmichael JD, Welkowitz J, Ferris S, Reitz RE, Danoff A, Kleinberg DL (2004). Variability and reliability of single serum IGF-I measurements: impact on determining predictability of risk ratios in disease development. Journal of Clinical Endocrinology and Metabolism.

[36] Matthews DR, Hosker JP, Rudenski AS, Naylor BA, Treacher DF, Turner RC (1985). Homeostasis model assessment: insulin resistance and beta-cell function from fasting plasma glucose and insulin concentrations in man. Diabetologia.

[37] Snijder MB, Visser M, Dekker JM, Seidell JC, Fuerst T, Tylavsky F, Cauley J, Lang T, Nevitt M, Harris TB (2002). The prediction of visceral fat by dual-energy X-ray absorptiometry in the elderly: a comparison with computed tomography and anthropometry. International Journal of Obesity and Related Metabolic Disorders.

[38] Visser M, Pahor M, Tylavsky F, Kritchevsky SB, Cauley JA, Newman AB, Blunt BA, Harris TB (2003). One- and two-year change in body composition as measured by DXA in a population-based cohort of older men and women. Journal of Applied Physiology.

[39] Economos CD, Nelson ME, Fiatarone MA, Dallal GE, Heymsfield SB, Wang J, Yasumara S, Ma R, Vaswani AN, Russell-Aulet M, Pierson RN (1997). A multi-center comparison of dual energy X-ray absorptiometers: in vivo and in vitro soft tissue measurement. European Journal of Clinical Nutrition.

[40] Clasey JL, Bouchard C, Teates CD, Riblett JE, Thorner MO, Hartman ML, Weltman A (1999). The use of anthropometric and dual-energy X-ray absorptiometry (DXA) measures to estimate total abdominal and abdominal visceral fat in men and women. Obesity Research.

[41] McDermott AY, Shevitz A, Knox T, Roubenoff R, Kehayias J, Gorbach S (2001). Effect of highly active antiretroviral therapy on fat, lean, and bone mass in HIV-seropositive men and women. American Journal of Clinical Nutrition.

[42] Biller BM, Shevitz A, Knox T, Roubenoff R, Kehayias J, Gorbach S (2002). Sensitivity and specificity of six tests for the diagnosis of adult GH deficiency. Journal of Clinical Endocrinology and Metabolism.

[43] Gasperi M, Aimaretti G, Scarcello G, Corneli G, Cosci C, Arvat E, Martino E, Ghigo E (1999). Low dose hexarelin and growth hormone (GH)-releasing hormone as a diagnostic tool for the diagnosis of GH deficiency in adults: comparison with insulin-induced hypoglycemia test. Journal of Clinical Endocrinology and Metabolism.

[44] Melmed S, Kleinberg D, Larsen PR, Kronenberg HM, Melmed S, Polonsky KS (2003). Anterior pituitary. Williams Textbook of Endocrinology.

[45] Ghigo E, Aimaretti G, Gianotti L, Bellone J, Arvat E, Camanni F (1996). New approach to the diagnosis of growth hormone deficiency in adults. European Journal of Endocrinology.

[46] Stein CJ, Colditz GA (2004). The epidemic of obesity. Journal of Clinical Endocrinology and Metabolism.

[47] Qu XD, Gaw GI, Al Sayed MY, Cohan P, Christenson PD, Swerdloff RS, Kelly DF, Wang C (2005). Influence of body mass index and gender on growth hormone (GH) responses to GH-releasing hormone plus arginine and insulin tolerance tests. Journal of Clinical Endocrinology and Metabolism.

[48] Veldhuis JD, Iranmanesh A, Ho KK, Waters MJ, Johnson ML, Lizarralde G (1991). Dual defects in pulsatile growth hormone secretion and clearance subserve the hyposomatotropism of obesity in man. Journal of Clinical Endocrinology and Metabolism.

[49] Weltman A, Despres JP, Clasey JL, Weltman JY, Wideman L, Kanaley J, Patrie J, Bergeron J, Thorner MO, Bouchard C, Hartman ML (2003). Impact of abdominal visceral fat, growth hormone, fitness, and insulin on lipids and lipoproteins in older adults. Metabolism.

[50] Veldhuis JD, Iranmanesh A, Lizarralde G, Urban RJ (1994). Combined deficits in the somatotropic and gonadotropic axes in healthy aging men: an appraisal of neuroendocrine mechanisms by deconvolution analysis. Neurobiology and Aging.

[51] Snyder DK, Clemmons DR, Underwood LE (1988). Treatment of obese, diet-restricted subjects with growth hormone for 11 weeks: effects on anabolism, lipolysis, and body composition. Journal of Clinical Endocrinology and Metabolism.

[52] Giordano R, Aimaretti G, Lanfranco F, Bo M, Baldi M, Broglio F, Baldelli R, Grottoli S, Ghigo E, Arvat E (2005). Testing pituitary function in aging individuals. Endocrinology and Metabolism Clinics of North America.

[53] Ghigo E, Goffi S, Nicolosi M, Arvat E, Valente F, Mazza E, Ghigo MC, Camanni F (1990). Growth hormone (GH) responsiveness to combined administration of arginine and GH-releasing hormone does not vary with age in man. Journal of Clinical Endocrinology and Metabolism.

[54] Lanzi R, Manzoni MF, Andreotti AC, Malighetti ME, Bianchi E, Sereni LP, Caumo A, Luzi L, Pontiroli AE (1997). Evidence for an inhibitory effect of physiological levels of insulin on the growth hormone (GH) response to GH-releasing hormone in healthy subjects. Journal of Clinical Endocrinology and Metabolism.

[55] Melmed S (1984). Insulin suppresses growth hormone secretion by rat pituitary cells. Journal of Clinical Investigation.

